# Diversity as the Fuel of Theory: Demographic Biases in CHILDES and Its Commentaries

**DOI:** 10.1111/desc.70092

**Published:** 2025-11-10

**Authors:** Camila Scaff, Georgia Loukatou, Alejandrina Cristia, Naomi Havron

**Affiliations:** ^1^ Laboratoire de Sciences Cognitives et de Psycholinguistique, Département d’études cognitives, ENS, EHESS, CNRS PSL University Paris France; ^2^ Human Ecology Group Institute of Evolutionary Medicine, University of Zurich Zurich Switzerland; ^3^ Stanford University Stanford California USA; ^4^ School of Psychological Sciences University of Haifa Haifa Israel; ^5^ Center for the Study of Child Development University of Haifa Haifa Israel; ^6^ Institute of Information Processing and Decision Making University of Haifa Haifa Israel

1

We are grateful to the authors of the six commentaries on our analysis of demographic biases in the CHILDES database. The commentaries unanimously celebrate CHILDES’ contributions, as do we. CHILDES is a remarkable resource, foundational for cross‐linguistic and theoretical advances in child language research and still holding extraordinary untapped potential. MacWhinney and Snow (2025) and Christiansen and McCauley (2025) argue that CHILDES corpora, even if biased, have proven repeatedly to be theoretically important: either as “edge cases” for universal claims, or as proof‐of‐concept data. Christiansen and McCauley (2025) further propose a comparative approach in which computational models are tested with different grain sizes across languages and demographics to mitigate biases.

At the same time, the commentaries reaffirm our invitation to reflect on the effects of systematic demographic biases. Omane et al. (2025) highlight the near‐total invisibility of Africa, despite its immense linguistic and cultural diversity, reminding us that “normal” is defined differently across contexts: In much of Africa, multilingualism and allomaternal care are the rule rather than the exception. Even as one of the most diverse resources available, CHILDES still predominantly represents English‐speaking, urban, highly educated, nuclear, monolingual families.

Several commentators stress the importance of intersectionality with multilingualism as a clear example. As Omane et al. (2025), Gökşun and Aktan‐Erciyes (2025), and Marchman and Weisleder (2025) note, CHILDES bilingual corpora overrepresent high SES, Western, urban centers, whereas most of the world's bilinguals do not grow up in such environments. They emphasize that diversity must be pursued across intersecting dimensions (SES, urbanization, family structure), rather than treated separately. A fundamental question then, as presented by Kidd and Garcia (2025), is whether demographic variables merely influence trajectories (e.g., vocabulary size, rate of growth) or whether they affect the *mechanisms* of acquisition themselves.

Moving forward, progress will require systematic metadata collection, a move away from convenience sampling (Kidd and Garcia 2025), purposive sampling targeting underrepresented realities (Omane et al. 2025), and denser recordings that capture a broader slice of daily life (Marchman and Weisleder 2025). It will also require finding solutions to ethical challenges and the shortage of support and funding of institutional agencies, as well as developing heuristics for making the most informed decisions from the data that we can collect (Havron et al. 2022; Hellwig et al. 2023).

We can only thank Brian MacWhinney and Catherine Snow for their revolutionary initiative 40 years ago. As they note in their commentary, it is heartening to see how much progress has been made. Documenting and embracing humanity in its diversity is a task that now requires renewed commitment. At a time when diversity initiatives in science face cuts, and research access for marginalized groups is shrinking, it is crucial to remember that diversity is not a threat to theory but its fuel: Theories of language acquisition must therefore treat demographic variation not only as a contextual modifier but as a potential driver of mechanism‐level change. Variation illuminates the shared processes that make language learning a universal human achievement.

## Conflicts of Interest

The authors declare no conflicts of interest.

## Data Availability

Data sharing not applicable to this article as no datasets were generated or analyzed during the current study.
